# Widespread signatures of local mRNA folding structure selection in four Dengue virus serotypes

**DOI:** 10.1186/1471-2164-16-S10-S4

**Published:** 2015-10-02

**Authors:** Eli Goz, Tamir Tuller

**Affiliations:** 1 Department of Biomedical Engineering, Tel-Aviv University, Ramat Aviv 69978, Israel; 2 Sagol School of Neuroscience, Tel-Aviv University, Ramat Aviv 69978, Israel; 3 SynVaccine Ltd. Ramat Hachayal, Tel Aviv 6971039, Israel

**Keywords:** dengue virus, folding energy, viral genome, comparative viral genomics, secondary structure

## Abstract

**Background:**

It is known that mRNA folding can affect and regulate various gene expression steps both in living organisms and in viruses. Previous studies have recognized functional RNA structures in the genome of the Dengue virus. However, these studies usually focused either on the viral untranslated regions or on very specific and limited regions at the beginning of the coding sequences, in a limited number of strains, and without considering evolutionary selection.

**Results:**

Here we performed the first large scale comprehensive genomics analysis of selection for local mRNA folding strength in the Dengue virus coding sequences, based on a total of 1,670 genomes and 4 serotypes. Our analysis identified clusters of positions along the coding regions that may undergo a conserved evolutionary selection for strong or weak local folding maintained across different viral variants. Specifically, 53-66 clusters for strong folding and 49-73 clusters for weak folding (depending on serotype) aggregated of positions with a significant conservation of folding energy signals (related to partially overlapping local genomic regions) were recognized. In addition, up to 7% of these positions were found to be conserved in more than 90% of the viral genomes. Although some of the identified positions undergo frequent synonymous / non-synonymous substitutions, the selection for folding strength therein is preserved, and thus cannot be trivially explained based on sequence conservation alone.

**Conclusions:**

The fact that many of the positions with significant folding related signals are conserved among different Dengue variants suggests that a better understanding of the mRNA structures in the corresponding regions may promote the development of prospective anti- Dengue vaccination strategies. The comparative genomics approach described here can be employed in the future for detecting functional regions in other pathogens with very high mutations rates.

## Background

The dengue virus (DENV) is classified to the *Flavivirus *genus of the *Flaviviridae *family, which also consists of additional human pathogenic viruses such as Yellow Fever (YFV), West Nile (WNV), Japanese Encephalitis (JEV) and Tick Borne Encephalitis (TBEV), which cause potentially lethal diseases [1].

DENV transmission, primarily caused by infected *Aedes aegypti *and *Aedes albopictus *mosquitoes, has been vigorously emerging in a growing number of countries over the last decades. The infection caused by DENV is widely recognized as a major public health concern, being endemic in over 100 countries, with 50-100 million infections worldwide a year, leading to half a million hospitalizations with about 2.5 - 5% mortality rate [2,3]. Despite the overall increase in Dengue incidence, which turns the virus into a significant burden for the health systems of the affected countries, currently no specific antiviral treatments exist. Similarly, there are no approved DENV vaccines, although extensive research has been conducted in this area over the past few decades, resulting in several candidates in various stages of development [4].

Four different Dengue serotypes widely appear in nature. These serotypes are closely related and cause very similar diseases in humans, although it was suggested that in terms of sequence similarity they are no more similar than some different species of flaviviruses [5-8]. Within each serotype the genetic diversity is significantly more restricted, but still sufficient to produce distinct viral genotypes [9].

The DENV genome is a positive polarity single stranded RNA of approximately 11 kb. It contains a type I cap structure, located at its 5´-end, and lacks the polyadenylation at its 3´-end. The genome is composed of 3 distinct functional parts: the unique coding region, and two flanking untranslated regions (UTRs). The UTRs contain important structural and functional elements required for viral translation and replication. During the viral life-cycle, the coding region is translated into a single precursor polyprotein, which is eventually processed co- and post-translationally by cellular and viral proteases, to produce 11 mature proteins [1].

Three Dengue structural proteins, C (capsid), M (membrane), E (envelope), serve as the main parts of the virion architecture and determine many of its morphological and functional properties; other seven nonstructural proteins, NS1, NS2A, NS2B, NS3, NS4A, NS4B and NS5, are involved as enzymes and/or other regulatory factors in different stages of the viral life cycle [1,10,11]

DENV and other viruses undergo a rapid evolutionary selection to evade the host immune system, and to efficiently compete with endogenous transcripts of the host cell over the gene expression machinery. Mechanisms that facilitate an efficient and selective viral replication are inherent in the nucleotide composition of the viral genomic sequence itself, and can involve the recruitment and/or modification of specific host factors. Non-synonymous mutations which alter the amino acid sequence provide a distinct evolutionary advantage due to selective pressure, allowing viruses to escape from innate defense mechanisms and acquired immune surveillance of the host, and to rapidly adapt to new cell types, tissues, or species. Yet, genomes (and even coding sequences), both viral and of other organisms, not only code for protein products but also carry additional information encrypted in the composition of alternating codons [12-19]. Being related to different biophysical and evolutionary characteristics, this information can be induced by synonymous mutations which preserve the underlying protein and may play an important regulatory role in different viral replication stages.

One specific feature of the genetic material (RNA or DNA) which is encoded in synonymous (and non-synonymous) aspects of its nucleotide composition is its structure/conformation which is formed by the molecule folding upon itself as a result of hydrogen bonds (and other biochemical connections) acting between pairs of nucleotides. The number and strength of these bonds determine the minimum free folding energy (MFE), which is related to the folding strength of a structure: more negative MFE indicates possibly stronger and more stable folding, while less negative MFE corresponds to weaker and less structured conformations. Extensive work has been directed to study mRNA structural elements in the 5' and 3' UTRs of DENV genomes revealing their critical role for regulation of translation and replication in host cells [10,11]. Yet, less attention so far has been paid to investigating the folding within the *coding regions*: previous works involving DENV [20-24], as well as other viruses [25-32] and organisms [13,33-36] have revealed some evidences for important functional secondary structures therein; however, they usually focused either on the viral untranslated regions or on very specific and limited regions at the beginning of the coding sequences, in a limited number of strains, and without considering evolutionary selection.

These and other studies suggest that besides the information determining the amino acid sequence, DENV coding regions may also encode additional important signals associated with the folding strength of structural elements, which can be involved in different steps of the viral life cycle.

In the current study we present, for the first time, a comprehensive, large scale, comparative genomics analysis of DENV *coding regions*, based on hundreds of different viral genomes from each of the four serotypes, aiming to identify significant signals encoded by synonymous information which are related to mRNA folding. These signals may indicate the presence of *local *evolutionary conserved cis-regulatory elements possibly performing important functions in different regulatory processes via their effect on various stages of gene expression, such as translation, RNA editing, RNA replication, and transcript degradation, and play a crucial role in viral efficiency and fitness.

## Results

**Figure 1:**
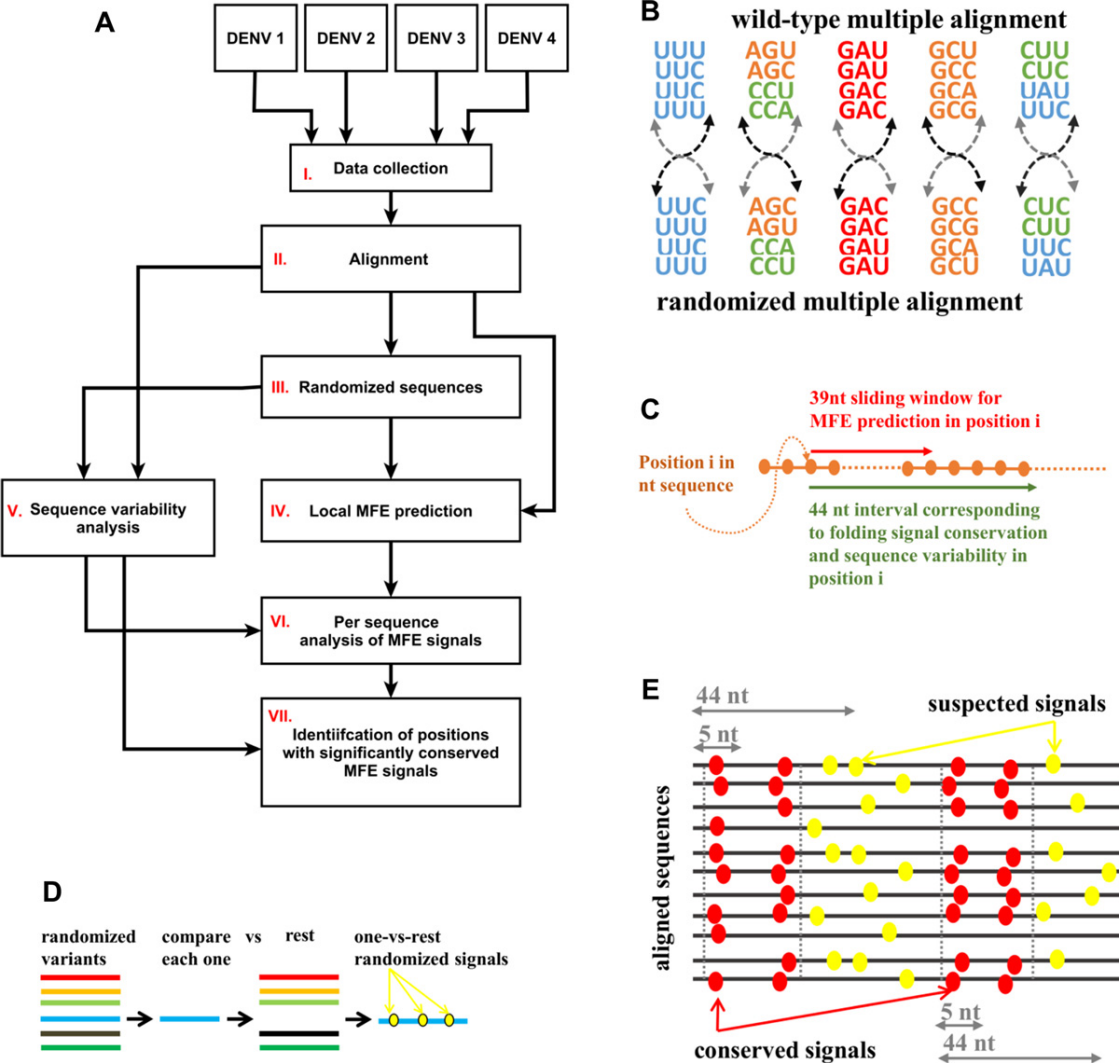
**A. Flow diagram of the study**. Our study includes the following general steps (details are in the main text): I. Coding regions of 1,670 Dengue genomes from 4 different serotypes were collected. II. The coding regions were aligned. III. Each of the wild type sequences was randomized 1000 times based on two different randomization models (evolutionary, and dinucleotide constrained). IV. Local minimum free folding energy (MFE) profiles were predicted for each wild type and randomized sequences separately. V. Profiles of sequence variability along the aligned coding regions were computed. VI. Wild type and randomized MFE profiles were compared to identify positions suspected to have a strong/weak local folding signal (p-value < 0.05). VII. Positions with MFE signals significantly conserved across different viral variants were identified. **B. Evolutionary-constrained randomization model **- synonymous codons in each column in the multiple alignment were permuted; if more than one amino acid was present (different colors) the permutations were restricted to the corresponding sets of synonymous codons. **C. Prediction of MFE in 39 nt windows (red arrow) along the coding sequence (brown)**; green arrow - 44 nt sequence interval corresponding to signal conservation and sequence variability analyses (the size of the interval was determined by the MFE prediction window size + allowed shift in signal position in conservation analysis). **D. One-Versus-Rest (OVR) model **- in each randomized variant, randomized MFE signals were identified by a position-wise comparison to the rest of the randomized variants from the same wild-type origin. **E**. **Signals conservation **- suspected MFE related signals (yellow) were defined as conserved if they appear in a significantly high (p-value < 0.001 with respect to randomized conservation levels based on OVR randomized signals) number of different sequences within a 5nt vicinity to each other (red). Two different clusters, each one consisting of two positions with a conserved MFE related signal are illustrated (distinguished by vertical dot lines); by definition, positions belong to the same cluster if they correspond to 44 nt length partially - overlapping genomic windows.

The different general stages of the study appear in Figure 1 (see Methods for more details):

1,670 coding regions of different genomes from four DENV serotypes were downloaded and aligned (Figure 1A I - II).

For each coding region we generated reference sets of 1000 randomized variants that maintain some of the fundamental properties of the original sequences (Figure 1A III) as follows:

To assess accurately the statistical significance of the predicted folding energies we employed a reference model that promises that the reported results cannot be explained by the amino acid composition of the encoded proteins and/or the evolutionary, phylogenetically dependent pressure on synonymous codons along the coding regions (evolutionary-constrained model). To this aim, we designed randomized variants (a Null model) that preserved both the amino acids order of the wild type sequences and the *column-wise *frequencies of synonymous codons at each position along their alignment (see more details in Figure 1B and in the Methods section).

In addition, to make sure that the obtained folding signals were not mainly a consequence of disrupted stacking base-pairs we compared our results with a randomization model designed to maintain both the encoded protein and the distribution of frequencies of pairs of adjacent nucleotides (dinucleotides-constrained model).

Local minimum free folding energy profiles *(*MFE-profiles) were computed for each wild-type and randomized sequence (Figure 1A IV, 1C).

To identify positions along the coding regions that were possibly selected during the course of viral evolution for significantly strong/weak folding (more/less negative MFE), we investigated the position-wise statistical differences between the MFE-profiles corresponding to the wild type sequences and MFE-profiles of their randomized variants (Figure 1A VI). For each sequence we considered the "suspected" positions for which the MFE values were found to be lower/higher than in 5% of the corresponding randomized variants (i.e. positions with empiric MFE associated p-value < 0.05) and analyzed their tendency to maintain the folding related signals across different viral strains (Figure 1A VII, C, E); in addition the role of sequence variability in this phenomenon was investigated (Figure 1A V).

To assess the expected number of suspected positions in randomized variants we designed the following procedure, named One-Versus-Rest (OVR) model: in each randomized MFE-profile, the suspected folding related signals were identified by a position-wise comparison to the rest of the randomized MFE-profiles from the same wild-type origin (Figure 1D). Conceptually, the average number of randomized suspected positions (MFE associated p-value < 0.05) obtained in this procedure evaluates the expected number of false positive signals and therefore can serve for an empirical false discovery rate estimation.

In addition, the suspected positions identified in randomized variants (randomized suspected positions) were used to obtain a null model for MFE signal conservation analysis.

Here we showed that all 4 DENV serotypes are likely to undergo an evolutionary selection on synonymous positions related to the local folding strength in numerous regions along their coding regions. We demonstrated that in these positions, MFE related signals tend to be conserved across different viral variants. By comparing with evolutionary and dinucleotide constrained background models we testified that the discovered signals are statistically significant and are not likely to be attributed to factors such as mutation bias of amino acid composition in the investigated sequences. We also showed a very low/not significant correlation between the levels of MFE related signal conservation and corresponding sequence variability values, demonstrating that conserved selection for local folding strength across different genotypes cannot be trivially explained by lowly-variable patterns in the synonymous codons and/or nucleotides preferences.

In the following sections we present a detailed analysis of our findings. A comprehensive description of the methods employed in the study can be found in the Methods section.

**Evidence that the DENV coding regions contain hundreds of positions that are likely to be selected for conserved strong or weak local folding structures**. Folding energy was estimated in all genomic windows of length 39 nt (motivated by an approximated average ribosomal footprint [37] and in the order of magnitude of various intracellular complexes [38] and functional mRNA structures [10,39] ) within the coding region of each viral genome, and the resulting values were used to construct local MFE*-*profiles: each position in a profile contained an MFE value computed in a window starting at this position.

MFE-profile of each wild-type sequence was compared in a position-wise manner to the MFE-profiles of the corresponding evolutionary-constrained randomized variants (randomized MFE profiles); positions with p-value < 0.05 were defined as "suspected" to have significantly more/less negative MFE in comparison to random (i.e. carrying a "suspected" folding related signal).

At the second step, aiming at distinguishing signals that are due to mutation bias from signals that undergo an evolutionary selection, we went further to identify positions along the coding region which tend to maintain MFE related signals in *different *viral variants. Such positions may belong to the same orthologous functional elements (i.e. elements conserved in various genomes with respect to their function but not necessarily conserved with respect to their sequence) and could have important implications for viral fitness.

To quantify the tendency of a particular position in the coding region to maintain a conserved signal, we computed the percentage of *different *sequences for which at least one suspected folding related signal was identified within a 5 nucleotides neighborhood of this position (Figure 1C, E). For convenience we termed this measure Folding Signal Conservation Index (FSCI). The FSCI values range between 0 (none of the sequences have any local MFE signal around the position) and 1 (100% of the sequences have a MFE signal within the allowed neighborhood).

To assess the statistical significance of MFE signal conservation, we compared the wild-type FSCI values to a reference model based on 1000 randomized alignments in which selection conservation was computed with respect to the randomized suspected signals detected via the OVR procedure. As a result, we identified positions with a statistically significant MFE signal conservation (FSCI associated p-value < 0.001; Benjamini-Hochberg false discovery rate 0.001); those of them with conservation levels higher than 0.20, 0.19, 0.21, 0.42 (thresholds which are equal to the maximal FSCI values achieved in random in serotypes 1 - 4 correspondingly for both folding signal directions) were defined as positions that are likely to undergo a *conserved *evolutionary selection for strong / weak folding (shortly, MFE-selected positions).

**Figure 2:**
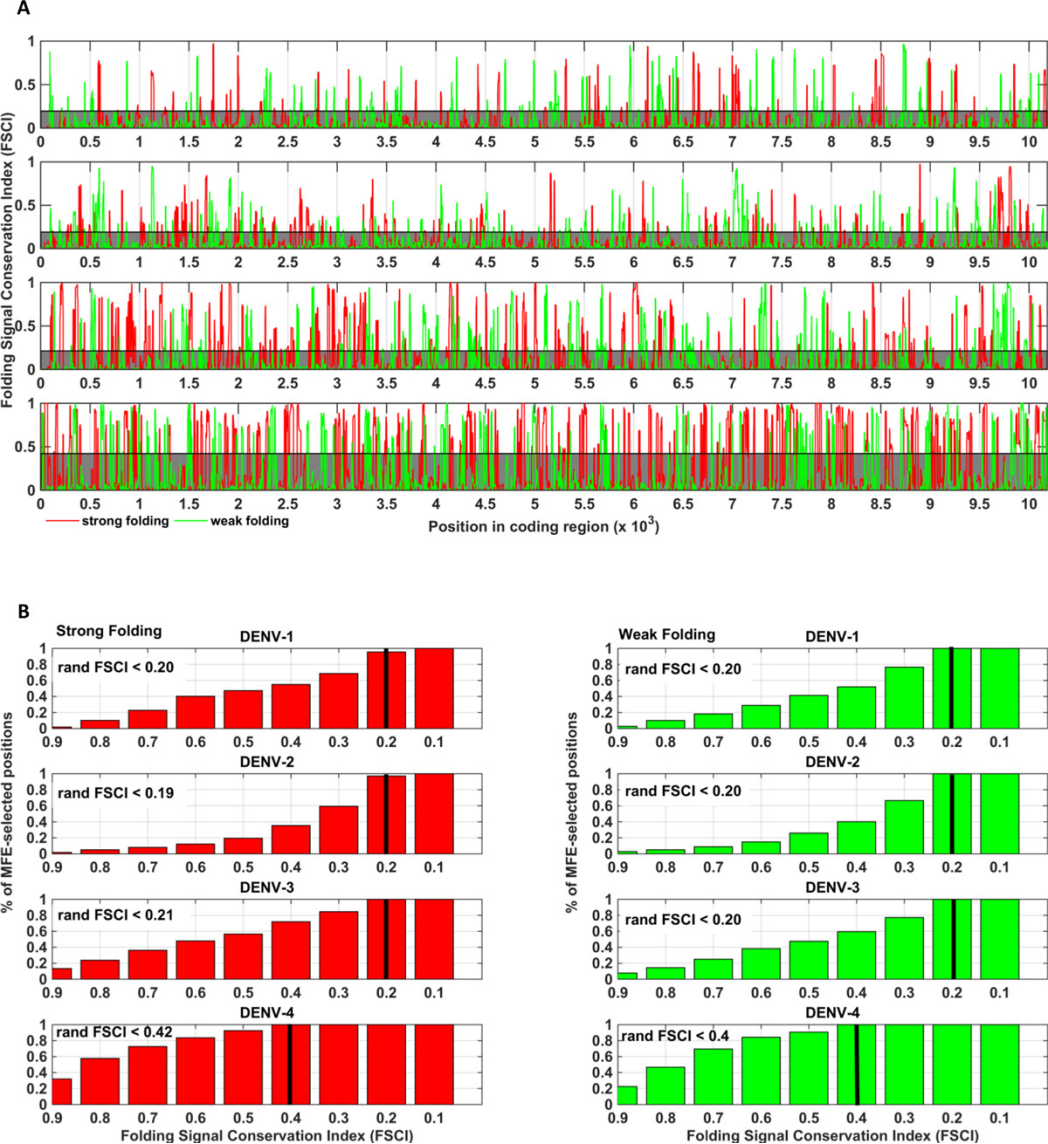
**A. Profiles of MFE related signal conservation along the coding regions of 4 DENV serotypes for strong (red) and weak (green) folding**. Positions with FSCI higher than a maximal value achieved in random (which is denoted by the shadowed area and is very similar for strong and weak folding) are not expected to be obtained by chance (p-value < 0.001 with respect to FSCI values based on randomized signals; Benjamini-Hochberg FDR = 0.001) and are defined as positions which may undergo a conserved selection for strong / weak local folding energy (shortly, MFE-selected). **B**. **Distribution of FSCI values in MFE-selected positions for strong / weak folding in 4 serotypes**. The maximal FSCI values achieved in random are explicitly annotated (rand FSCI) and marked by black vertical bars. Total number of MFE-selected positions in wild-type is 40-100 folds higher than in random.

Profiles of FSCI values along the coding regions are shown in Figure 2A. Positions with a significantly conserved strong folding signal were found to constitute 53, 65, 62, 66 different clusters in serotypes 1 - 4 correspondingly; likewise, weak local folding signal was identified as conserved in positions grouped in 49, 73, 58, 65 clusters. Each cluster was comprised of positions with significantly conserved MFE related signals predicted in intersecting 44 nt genomic windows (39 nt folding window size + 5 nt allowed shift in signal position in conservation analysis); these positions could be possibly attributed to the same or partially-overlapping folding elements. Analysis of enrichment of specific DENV genes with clusters of positions with a significant conservation of folding energy signals and distribution of these clusters over different genes can be found in Additional file 1.

The resulting conservation levels were found to be spread over a wide range of values; specifically 20%-90% of MFE-selected positions (depending on serotype and the direction of the folding signal) possessed FSCI values greater than 0.5 (meaning that the MFE related signals in these positions were maintained in more than 50% of the sequences); in 2%-7% of MFE-selected positions the conservation levels where higher than 0.9 (meaning a conservation of the MFE signal therein in more than 90% sequences; Figure 2B).

**Figure 3:**
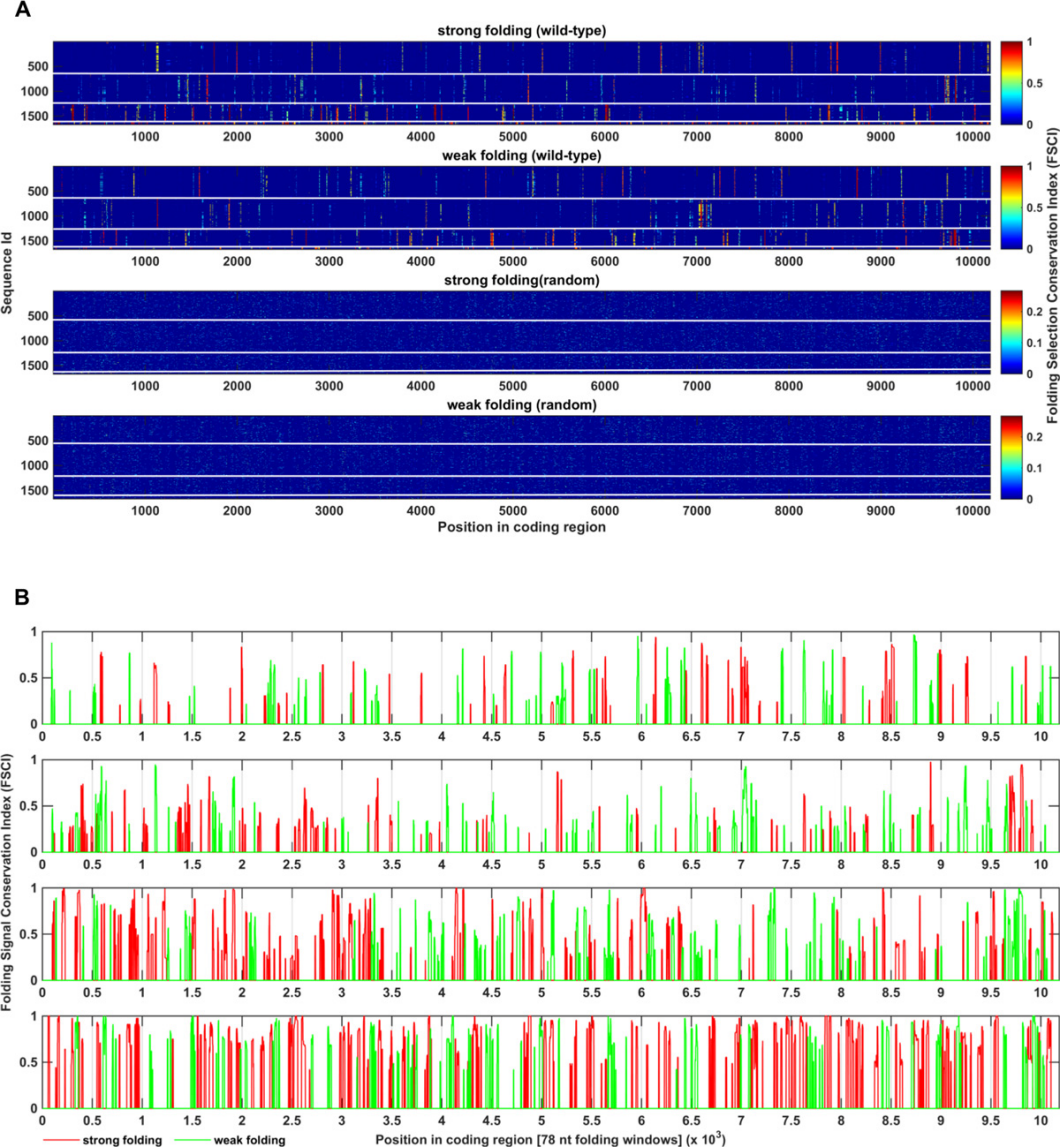
**A. Selection matrices for strong / weak folding for wild-type and one corresponding randomized variant in 4 DENV serotypes**. Each row in the matrix corresponds to one sequence; columns are positions along the coding region. If sequence i has a suspected MFE related signal (p-value < 0.05) in position j, the entry (i,j) has a value equal to the corresponding FSCI; otherwise it is equal to zero. White horizontal lines separate between sequences belonging to different serotypes (serotypes are ordered from top to bottom, i.e. sequences 1-652 belong to serotype 1; 653-1268 to serotype 2; 1269-1625 to serotype 3 and 1626-1670 to serotype 4). The randomized matrices are comprised of a single randomized variant for each sequence. We can clearly distinguish positions with conserved MFE related signals with different conservation levels in the wild-type, contrasting with a white noise resembling appearance in the randomized variants. **B**. **Robustness of folding signal conservation to folding window size**. MFE related signal conservation basing on 39nt folding windows is shown in positions that overlap with MFE conserved signals basing on 78 nt windows along the coding regions of 4 DENV serotypes for strong (red) and weak (green) folding. As many as 80%, 77%, 90%, 92% of conserved signals related to strong folding (red) and 80%, 82%, 85%, 80% conserved signals related to week folding (green) (for serotypes 1 - 4 correspondingly) overlapped with MFE conserved signals identified with respect to a two times larger folding window (78 nt); this overlap was found to be on average 2.5 - 3 folds higher than in random which was based on conservation levels (with respect to 78 nt folding window) in 100 randomized alignments (p-value < 0.01).

The total amounts of MFE-selected positions in all serotypes were found to be significantly higher (p-value < 0.001; on average 40-100 folds, depending on serotype and the direction of the folding signal) than those obtained in the randomized variants (Figure 3A). Moreover, as was stated above, the maximal FSCI value achieved in random is 0.2-0.42 while in wild-type 35%-100% of MFE-selected positions possessed higher conservation levels (depending on serotype and the direction of the folding signal).

Finally, to estimate the robustness of our results with respect to the folding window size we calculated the MFE-selected positions basing on sliding windows twice as large as initially (78 nt instead of 39 nt). As a result, we found that as many as 80%, 77%, 90%, 92% of significantly conserved signals related to strong folding and 80%, 82%, 85%, 80% of significantly conserved signals related to weak folding (basing on 39 nt folding window size) overlapped with conserved signals identified with respect to 78 nt folding window size (Figure 3B), and this overlap was found to be on average 2.5 - 3 folds higher than in random which was based on conservation levels (with respect to 78 nt folding window) in 100 randomized alignments (p-value < 0.01; see also Methods section).

**Conserved selection for strong / weak folding related signals cannot be explained basing only on dinucleotide composition**. Arguably, the dinucleotide content is important when assessing the predicted free energy of RNA secondary structures [40-42]. In particular, it was suggested that disruption of naturally occurring biases in dinucleotide frequencies in genomic sequences of different organisms have been common sources of erroneous conclusions in previous studies [42,43]. To make sure that the presence of excess local secondary structure in coding regions of mRNA is not merely an artifact resulting from the failure to control for dinucleotide composition we verified the robustness of our findings by analyzing a dinucleotide-constrained randomization model controlling for the distribution of dinucleotide frequencies (see Methods section).

**Figure 4:**
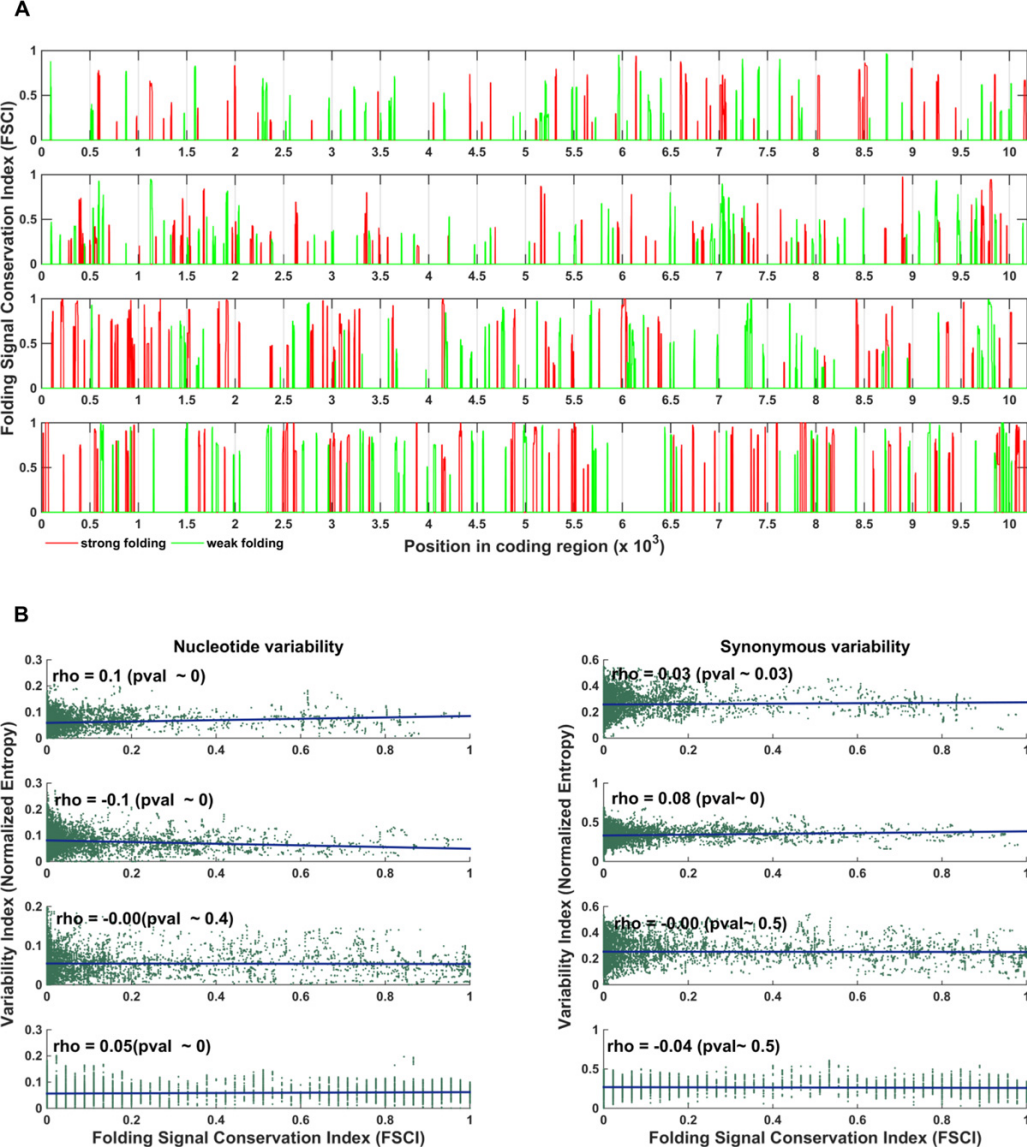
**A. Conserved selection for strong / weak folding related signals cannot be explained basing only on dinucleotide composition**. MFE related signal conservation with respect to evolutionary-constrained randomization model is shown in positions that overlap with MFE conserved signals with respect to dinucleotide-constrained randomization model along the coding regions of 4 DENV serotypes for strong (red) and weak (green) folding. As many as 60%, 52%, 49%, 34% of positions with conserved signals related to strong folding (red) and 62%, 58%, 43%, 44% of positions possessing weak folding signal conservation (green) (for serotypes 1 - 4 correspondingly) overlapped with MFE conserved signals identified with respect to dinucleotide-constrained randomization model, and this overlap was not likely to appear in random (p-value < 0.001; no overlap was observed in 1000 randomized variants). **B. The regions with significantly conserved strong / weak folding signal cannot be explained based only on sequence conservation**. A low / insignificant Spearman correlation between conservation levels of MFE related signals (x-axis) and the nucleotide / synonymous variability (y-axis) in the corresponding genomic intervals is demonstrated. The least square linear approximation of the data points is plotted in blue.

We found that as many as 60%, 52%, 49%, 34% of positions with significantly conserved signals related to strong folding and 62%, 58%, 43%, 44% of positions possessing weak folding signal conservation (identified with respect to evolutionary-constrained model for serotypes 1 - 4 correspondingly) overlapped with MFE conserved signals identified with respect to dinucleotide-preserving randomization model (Figure 4A), and this overlap was not likely to appear in random (p-value < 0.001 basing on conservation levels in 1000 randomized alignments; specifically, *no overlap *was observed in the case of the randomized genomes).

This result is further supporting the conjecture that dinucleotides alone cannot explain the majority of obtained MFE signals identified with respect to the evolutionary-constrained model, and thus at least some of them undergo a conserved evolutionary selection for strong/weak folding and are not just artifacts of disrupting natural occurring biases in pairs of adjacent nucleotides.

**The regions with significantly conserved strong / weak folding signals cannot be explained based only on sequence conservation**. Although the nature of the evolutionary-constrained model excludes the possibility of significant MFE signal conservation in regions with a low sequence variability across different viral variants (in such case the randomization will not have enough degrees of freedom to produce a sufficient variety of variants for a reliable statistical analysis) we decided to additionally explore the plausibility that conservation of folding signals may be a 'side effect' of a conserved nucleotides composition or a preference for specific synonymous codons (due to reasons not directly related to folding).

To this aim we quantified the variability among different sequences along the coding region, once with respect to a preference for synonymous codons and once with respect to nucleotides content, by considering an entropy based measure in each position in the coding region (see Methods); this measure returns a value which ranges between 0 (no variability; i.e. a preference for a certain nucleotide / synonymous codon ) and 1 (maximal variability; i.e. a uniform usage of all nucleotides / synonymous codons).

To assess the relationship between the conservation levels of MFE related signals and sequence variability therein, we calculated Spearman correlations between: 1) the signal conservation profiles and 2) the nucleotide / synonymous variability profiles constructed by locally averaging the corresponding variability values in all 44nt genomic intervals (the size of the intervals was chosen to match the 39 nt local windows in which the MFE was predicted + the allowed 5nt position shift in signal conservation analysis; see the Methods section and Figure 1C); we also calculated, in a similar manner, the correlations between 1) the signal conservation profiles and 2) the variability profiles which were normalized with respect to their randomized variants (based on 1000 randomized alignments) to obtain z-score values (see Methods).

We found that the correlation between the MFE signal conservation, and nucleotide and synonymous variability / z-score normalized variability is too low to conclude that regions with lower variability tend to have higher tendency for MFE signal conservation. Specifically the correlation values were found to be confined in a narrow [-0.1 0.1] interval around zero for different types of variability profiles (Figure 4B); i.e. less than 10% of the variance in signal conservation variable can be explained by the variability values.

These results support the conjecture that the conservation of MFE related signals is not necessarily and only due to a preference of specific synonymous codons or conserved nucleotide content, and cannot be solely explained by the low sequence variability, thus supporting the evidence for a direct, conserved selection on positions for strong / weak folding.

## Discussion

In this study, we analyzed the coding sequences of 1,670 genomes in four different DENV serotypes in order to identify regions that are selected for strong or weak mRNA folding, and thus are possibly involved in the regulation of different stages of the viral replication cycle.

We suggested that positions along the coding region with a significant conservation of folding associated signals are grouped in 49 - 73 clusters related to partially overlapping local genomic regions that may tend to undergo an evolutionary selection for a strong or weak folding. In addition, in certain cases up to 7% of these positions were found to be conserved in more than 90% of the sequences.

We showed that positions with significantly conserved strong/weak folding signals are not likely to appear in random, thus suggesting that they were possibly selected in the course of viral evolution to fulfill certain biological functions. We also demonstrated that the identified folding signals overlap significantly with signals obtained by doubling the sliding window supporting the conjecture that the results presented in this study are robust to the size of local genomic regions in which the minimum free folding energy is predicted.

The statistical significance of the selection for strong/weak folding was tested with respect to an evolutionary constrained null model that maintains the mutational bias and protein content of the viruses; in addition the possibility that the obtained signals were mostly due to disruption of naturally occurring biases in pairs of adjacent nucleotides was rejected by a comparison to the dinucleotide-constrained randomizations. Both randomization models were designed to preserve the essential organizational principles of genomic sequences including certain non-random features and naturally occurring biases. It should be noticed that these 'non-random' properties of the randomization models does not contradict the fact that the corresponding randomized sequences may have strong/weak folding in some arbitrary regions; however, these false positive signals are expected to be filtered out in signal conservation analysis described above.

Finally, we found no significant correlation between the levels of sequences variability and the levels of conservation of MFE signals, supporting the conjecture that positions with a conserved selection for strong/weak folding may be related *directly *to biophysical attributes of gene expression regulation and/or viral replication via folding. This result also means that the reported conserved MFE related signals are not necessarily a consequence of other (non-direct) constraints imposed on specific sequence patterns, and/or formation of particular secondary structures. Nevertheless, we would like to emphasize that the analyses performed in this study neither cannot eliminate all alternative explanations for the emergence of the reported folding signals other than a direct evolutionary selection nor they are enough for a full understanding of the possible functional/evolutional properties of the folding strength along the viral mRNA. To this goal additional biological validations are required.

It is important to note that in our analysis we didn't require the preservation of the structure architecture itself, but analyzed a preference for folding strength while permitting unconstrained variability in the underlying secondary structure (e.g. [44] ). This approach was motivated by the fact that often the level of MFE embodied in the element, rather than the specific structure itself, plays a significant role in the viral life cycle. This assumption is based on previous studies that have demonstrated that the local folding along the mRNA affects various aspects of gene expression; specifically, it was suggested that in certain contexts and positions along the transcript, both strong and weak mRNA folding can have positive effect on gene expression and organismal fitness [12,45,46]. Research on the conservation of specific RNA structures is deferred to future studies.

Rather than performing a global analysis of the entire mRNA (or very long mRNA sub-sequences), we predicted the folding energies in local windows along the genome. This approach was motivated by several considerations. To date, it is believed that the structured RNA elements identified as being involved in different regulatory processes are often discrete, and usually involve short-range Watson-Crick base-pairing which span no more than 100nt [47,48]. Such local structures are more likely to be formed in vivo in actively translating mRNAs. Furthermore, in a cell, most RNA molecules, and in particular messenger RNAs, interact with a wide variety of proteins, further limiting the predictability of long-range effects [47]. Moreover, there are specific cases of long range RNA-RNA interactions in Dengue [49]; however, usually the interactions between the RNA, and the replication and translation machinery are expected to prevent 'long range' RNA folding (due to its unfolding by proteins and factors related to these steps), but still enable generating local mRNA structures.

From the computational perspective, current folding prediction algorithms provide meaningful predictions from small-medium scale sequences, while long sequences suffer from a degrading accuracy [50]. Thus, in our opinion, the local folding framework, advocated both by computational and biophysical reasoning, enables a more accurate modeling of the dynamic, location dependent nature, of in vivo structure formations, while minimizing unfavorable computational inaccuracies and biases.

In contrast to previous works (e.g. [51,52]) which provided evidence for existence of RNA structures throughout (usually more than 150 nt long) genomic fragments, our local approach also enables a high resolution identification of positions selected for strong/weak folding energy. Moreover, our comprehensive statistical analysis based on a carefully chosen randomization model discussed above, enables to target evolutionary selected regions where the selected positions are significantly conserved across different viral strains.

As we have already noticed before, previous studies have discovered several structural elements at the 5' end of the capsid gene (at the beginning of the coding region) and proposed that they take part in replication and translation regulation [20-24]. It is important to emphasize that since the underlying sequences are relatively conserved these regions were not detected by the proposed evolutionary-constrained randomization since in this case permutation of lowly-variable columns doesn't produce enough variability in randomized variants. Nevertheless, this fact doesn't derogate from the ability to identify many additional/novel folding elements with a variable underlying sequence suggested to be under selection related directly to their possibly functional mRNA folding.

## Conclusions

In this work, we presented a large scale comparative genomic analysis based on hundreds of viral variants in each one four different DENV serotypes. We suggested that positions along the coding region with a significant conservation of folding associated signals are grouped in 49 - 73 clusters related to partially overlapping local genomic regions that may tend to undergo an evolutionary selection for a strong or weak folding. We showed that the reported signals of conserved MFE selection cannot be explained basing on amino-acid, dinucleotide, or mutational bias only and are not necessarily a consequence of other (non-direct) constraints imposed on specific sequence patterns, and/or formation of particular secondary structures. We believe that further molecular biology studies guided by the computationally predicted positions may promote discoveries of novel regulatory mechanisms of the DENV and other viruses; some of them may help developing new vaccination strategies.

## Methods

**Data preparation**. 1,670 complete coding sequences of 4 DENVserotypes (651, 615, 356, 45 strains in serotypes 1 - 4 respectively) were downloaded.

We first translated the nucleotide coding regions and then aligned the resulting amino acid sequences by Clustal Omega package [53] with default parameters. To obtain the multiple alignment of corresponding nucleotide sequences we mapped the aligned amino acids back to the nucleotide sequences basing on the original nucleotide composition of each genome. Multiple alignment conservation scores can be found in Additional file 1.

A list of accession numbers corresponding to the analyzed genomes can be found in Additional file 2.

**Genome randomization models**. To investigate selection for folding strength, wild-type minimum free folding energy (MFE) values were compared with corresponding sequence-randomized controls which preserve certain nonrandom features of the naturally occurring sequences. To exclude the possibility that the obtained signals were simply due to amino acid selection pressure (i.e., selection on the protein sequence), as opposed to selection for the folding strength, we restricted our randomized variants to maintain the amino acids order and content (and thus the encoded protein), by sampling from the set of synonymous codons for each amino acid position. To model evolutionary constraints (not necessary related to folding) imposed on synonymous variability in different genomic positions (e.g. mutational bias) we maintained the distribution of synonymous codons (and thus nucleotides) for each *column *in the interserotype multiple alignment matrix (matrix containing aligned sequences of 4 serotypes). This was achieved by random permutations of synonymous codons for each column in the alignment matrix; in the case of multiple amino acids in a column, each amino acid was permuted separately (Figure 1B). This randomization method samples (with repetitions) uniformly and independently multiple alignment columns from the set of corresponding synonymous codons, thus generating for each amino acid the same 'vertical' codon frequencies as in the original alignment matrix (but in a different order).

To model the composition of nucleotide pairs which are argued to have an important effect on formation of secondary structures, a model that preserves both *the amino acids order and content*, and the frequencies distribution of 16 possible pairs of adjacent nucleotides (*dinucleotides*) for each sequence separately was used. Although efficient methods exist for preserving the amino acids (e.g. permutation of synonymous codons) or the dinucleotides content (e.g. random generation of an Euler path in a De Bruijn-like graph, whose edges represent the dinucleotides [54]) separately, it has been difficult to combine them for satisfying both of the constraints. To overcome these difficulties, we used an elegant algorithm proposed in [55] which is based on a multivariate Boltzmann sampling scheme, initially introduced in the context of enumerative combinatorics. This algorithm produces random variants which feature both correct dinucleotide frequencies and coding capacity while being generated with provably *uniform *probability. We used the original source code which can be found in http://csb.cs.mcgill.ca/sparcs.

For each one of 1,670 wild-type sequences, we computed 1,000 randomizations basing on each one of the randomization models, resulting in more than 3 million variants.

**Local minimum free folding energy profiles**. Minimum free folding energy (MFE) is a thermodynamic energy involved in maintaining a secondary structure available to perform physical work while being released, and thus is characterized by non-positive values. mRNA secondary structure is believed to be in the most stable conformation when minimum amount of free energy is exerted (the MFE obtains the most negative value).

The local minimum free folding energy profiles (MFE-profiles) were constructed by applying a 39 nt length sliding window to a genomic sequence (Figure 1C): in each step the MFE of a local subsequence enclosed by the corresponding window was calculated by Vienna (v. 2.1.9) package RNAfold function with default parameters [56]. This function predicts the minimum free folding energy and the associated secondary structure for the input RNA sequence using a dynamic programming based on the thermodynamic nearest-neighbor approach (the Zucker algorithm) [57-59].

**Minimum free folding energy significance test**. In order to assess the statistical significance of the folding strength in a particular position in a sequence, we compared the MFE values in this position with the MFE values in the corresponding position in each one of the randomized variants by calculating an empiric p-value - a proportion of the randomized values as extreme as in the wild type. Positions with MFE related p-value < 0.05 were defined as having a "suspected" MFE related signal; due to a high false discovery rate (Benjamini - Hochberg approach) of MFE signals in individual sequences we went further and compared the positions of suspected signals across different genomes

**Conservation of local folding signals**. Due to genetic variability on the one hand, and possible inaccuracies in sequencing and multiple alignment on the other, positions selected for a significant strong (weak) folding in different genomes may be shifted one with respect to the other. To account for these possible displacements when quantifying the conservation of MFE related signals across different sequences, we defined a Folding Signal Conservation Index (FSCI) at a particular position as a percentage of different aligned sequences which have at least one signal inside a 5 nt length genomic neighborhood of this position (Figure 1C, E). FSCI takes a range of values between 0 and 1: the higher the value - the more different sequences have MFE related signals inside the corresponding neighborhood (higher signal conservation), the lower - the less sequences have MFE related signals in common (lower signal conservation). A vector of FSCI values in all positions along the coding regions ( Folding Signal Conservation Profile) was calculated by applying a 5 nt sliding window to the matrix of aligned MFE- profiles (for each serotype and folding signal direction separately), and calculating at each step the corresponding signal conservation index.

Positions with significantly high MFE related signal conservation were identified by comparing the wild type FSCI values in each position to the FSCI values from the corresponding positions in 1000 randomized signal conservation profiles (generated basing on suspected signals identified in 1000 randomized alignments via the OVR model). Those positions with significantly conserved folding signals (p-value < 0.001 with respect to randomized selection conservation values, Benjamini-Hochberg false discovery rate = 0.001) which had conservation levels higher than achieved in all corresponding randomized variants were defined as positions that undergo a conserved evolutionary selection for strong/weak folding (MFE-selected positions).

The resulting positions are not independent: parts of them belong to intersecting genomic regions and could be possibly attributed to the same or partially-overlapping folding elements. Therefore we defined clusters of MFE-selected positions; each cluster consists of all positions with significant signal conservation such that the distance between two consecutive positions in a cluster is no more than 44 nt . According to this definition positions within a particular cluster correspond to partially - overlapping genomic windows (39nt folding window + 5nt offset used in signal conservation analysis); in contrast positions belonging to different clusters are thought as independent with respect to the performed local MFE analysis.

We emphasize that conservation of MFE related signals was analyzed for each serotype, and folding signal direction separately; specifically, in each case we accounted for positions selected for only one folding direction, either strong or weak. Moreover, the analysis of signal conservation was performed with respect to the evolutionary-constrained model only, since (in contrast to the dinucleotide-preservation or any other model based on a single sequence) it takes into consideration the co-evolution of viral variants and their phylogenetic dependencies.

**Robustness of folding signal conservation to folding window size**. To estimate the robustness of the MFE-selected positions with respect to the folding window size we repeated the analysis described above with folding windows twice as large as initially (78 nt instead of 39 nt). We then defined a robustness measure as the percentage of the 44 nt genomic regions corresponding to MFE-selected positions (based on 39 nt folding windows + 5nt offset used in signal conservation analysis) which have more than 50% overlap with at least one 83 nt genomic region corresponding to an MFE-selected position (based on 78 nt folding windows + 5nt offset conservation analysis offset).

**One-versus-rest (OVR) model**. In order to estimate the expected number of suspected MFE related signals (p-value < 0.05) in random and in order to generate a null model for estimating the statistical significance of MFE signal conservation in different positions, we simulated MFE suspected signals in randomized variants according to the following procedure named One-Versus-Rest (OVR) model: for each one of the N randomized variants corresponding to a specific wild-type sequence, we identified iteratively its MFE-related suspected signals with respect to the rest of the N-1 random variants (Figure 1D). We then used the obtained sets of the randomized MFE signals to construct the randomized signal conservation profiles: each randomized profile was generated by picking (without repetition) a single one-versus-rest randomized set of selected positions for each wild type sequence (resulting in a randomized alignment variant) and then applying the methodology for computing signal conservation levels as described above.

**Normalized entropy as a measure for sequence variability**. We defined the nucleotide / synonymous variability at a position i in the nucleotides / protein multiple alignment as

Shannon entropy of a distribution on nucleotides / synonymous codons corresponding to the consensus amino acid, normalized by the maximal possible entropy value possible in the given position (this measure was also, independently, introduced, in [60] ):

$$V_{i}=-\frac{\overset{n}{\underset{j=1}{\sum }}p_{j}{\text{log}}_{2}\left(p_{j}\right)}{{\text{log}}_{2}n}$$

here n is the number of distinct elements in the corresponding alphabet,; and p_j _are their relative frequencies (in the case of nucleotide variability, n = 4, i.e. the number of different possible nucleotides; for synonymous variability n is the number of different synonymous codons corresponding to the consensus amino acid in this position).

This variability measure takes values between 0 and 1, and describes how dispersed the distribution of the alphabet elements is: higher values correspond to more uniform nucleotide / codon usage; lower values correspond to more biased nucleotide / codon usage, indicating that some nucleotides / synonymous codons are preferred.

The variability measure was computed for each serotype separately. The synonymous variability index was computed based on the consensus amino acid (the most frequent amino acid) in each position in the multiple alignment. In order to neutralize biases due to poor high number of indels and low consensus values( high amino acid variability), we filtered out positions with consensus levels of less than 90%, and number of gaps of more than 10% (resulting in ~ 4%, 6%, 3%, 3% filtered positions in serotypes 1 - 4 respectively). In addition, positions corresponding to singleton amino acids Methionine and Tryptophan (with a natural absence of variability) were excluded.

The variability profiles were constructed by applying a 44 nt sliding window along the alignment and averaging at each step the nucleotide / synonymous variability values at positions within the corresponding window. The window size was defined in a way that each such window matches the 39 nt genomic region in which the folding for the corresponding positions was predicted + a 5nt allowed shift used in MFE signals conservation analysis (Figure 1C).

The z-score normalized synonymous variability was constructed by computing in each position a z-score with respect to 1000 variants based on randomized multiple alignments (each randomized alignment was constructed by taking a single, amino acids order preserving, random variant of each wild-type genome):

$$V_{z-score}=\frac{V-\mu }{\sigma }$$

(μ / Ϭ - mean / s.t.d of randomized variability values at a particular position).

**Software**. Multiple alignments were performed with Clustal Omega package (v.1.2.0). Folding energies were predicted with RNAfold function from Vienna package (v.2.1.9) adapted by us to work with sliding windows. Other computations were performed using Matlab^® ^software (MathWorks Inc.). For high performance computing, a Linux based cluster system was employed.

## List of abbreviations

DENV - Dengue Virus; MFE - Minimum Free Folding Energy; FSCI - Folding Signal Conservation Index; OVR - One versus Rest; FDR - False Discovery Rate.

## Competing interests

A patent application describing this work was submitted. The authors declare that they have no other competing interests.

## Authors' contributions

EG and TT analyzed the data and wrote the paper

## Supplementary Material

Additional File 1 **supplementary_analysis.docx**. This file includes the following additional analyses: Multiple alignment conservation scores; Enrichment of DENV genes with clusters of positions with a significant conservation of folding energy signals and distribution of these clusters over different genes. Click here for file

Additional File 2 **denv_accession_numbers.csv**. This file includes GenBank accession numbers for sequences analyzed in this study Click here for file

## Declarations

EG is supported in part by a fellowship from the Edmond J. Safra Center for Bioinformatics at Tel-Aviv University. TT is partially supported by the Minerva ARCHES award.

The publication costs were funded by Tel Aviv University resources.

This article has been published as part of *BMC Genomics *Volume 16 Supplement 10, 2015: Proceedings of the 13th Annual Research in Computational Molecular Biology (RECOMB) Satellite Workshop on Comparative Genomics: Genomics. The full contents of the supplement are available online at http://www.biomedcentral.com/bmcgenomics/supplements/16/S10.
