# Supplementary material for: Widespread signatures of local mRNA folding structure selection in four Dengue virus serotypes
Source: BMC Genomics. 2015 Oct 2;16(Suppl 10):S4. doi: 10.1186/1471-2164-16-S10-S4 (PMC4602183; doi:10.1186/1471-2164-16-S10-S4)
Supplement: Additional File 1 — supplementary_analysis.docx. This file includes the following additional analyses: Multiple alignment conservation scores; Enrichment of DENV genes with clusters of positions with a significant conservation of folding energy signals and distribution of these clusters over different genes. [file 1471-2164-16-S10-S4-S1.pdf]

Widespread signatures of local mRNA folding structure selection in four Dengue virus serotypes

Supplementary

Eli Goz [1] (guzeliez@post.tau.ac.il), Tamir Tuller [1,2] (tamirtul@post.tau.ac.il)

Department of biomedical engineering, Tel-Aviv University, Israel[1]; Sagol School of Neuroscience, Tel-Aviv, University, Israel[2]

Multiple alignment conservation scores

Multiple alignment conservation score was defined by us as an average sum-of-pair score (SP). For the i-th column in the alignment we define $P_{ijk}=1$ for every pair A_{ij} and A_{ik} of elements (either nucleotides of amino acids, depending on the type of the aligned sequences) which are equal to each other and $P_{ijk}=0$ otherwise. The score Si for the ith column is

$$S_i = \frac{1}{N(N-1)/2}\sum_{j=1}^{N}\sum_{k=j+1}^{N} P_{ijk}$$

and the SP for the alignment is:

$$SP = \frac{1}{M}\sum_{i=1}^{M} S_i$$

The following table summarize the SP scores for multiple alignments of amino-acids SP(AA) and corresponding nucleotides sequences SP(NT) for each serotype:

	**SP (AA)**	**SP(NT)**
**DENV1**	0.98	0.95
**DENV2**	0.97	0.94
**DENV3**	0.98	0.95
**DENV4**	0.96	0.94

Table T1. Multiple alignment average sum-of-pair score (SP) for amino acid (the first column) and nucleotide (the second column) sequences in each serotype.

A list of GenBank accession numbers for the analyzed sequences can be found in the Additional file 2).

Enrichment of DENV genes with clusters of positions with a significant conservation of folding energy signals and distribution of these clusters over different genes.

Definition of regions coding for specific DENV genes were taken from annotated reference genomes: NC_001477, NC_001474, NC_001475, NC_002640 for serotypes 1 – 4 correspondingly. The genes coordinates (start, end) were than mapped to the aligned genomes.

The distribution of clusters over different DENV genes is shown in tables T1 and T2.

	ancC	prM	E	NS1	NS2a	NS2b	NS3	NS4a	NS4b	NS5
**DENV1**	0	0.0377	0.1887	0.0943	0.0377	0.0755	0.1887	0.0566	0.1132	0.2075
**DENV2**	0.0308	0.0462	0.1538	0.1231	0.1077	0.0462	0.1538	0.0308	0.0769	0.2308
**DENV3**	0.0323	0.0645	0.1452	0.0806	0.0806	0.0323	0.1774	0.0322	0.0645	0.2903
**DENV4**	0.0606	0.0606	0.1515	0.0606	0.0606	0.0303	0.2121	0.0607	0.0758	0.2273

Table T1. Percentage of clusters of positions with a significant conservation of strong folding signals in different genes

	ancC	prM	E	NS1	NS2a	NS2b	NS3	NS4a	NS4b	NS5
**DENV1**	0.0612	0.0408	0.1020	0.1633	0.0408	0.0204	0.2245	0.0204	0.0612	0.2653
**DENV2**	0.0274	0.0548	0.1507	0.0822	0.0548	0.0411	0.1781	0.0411	0.0822	0.2877
**DENV3**	0.0345	0.0690	0.1379	0.1034	0.0862	0.0345	0.1552	0.0517	0.0690	0.2586
**DENV4**	0.0154	0.0462	0.1692	0.1231	0.0769	0.0308	0.1538	0.0462	0.0769	0.2462

Table T2. Percentage of clusters of positions with a significant conservation of weak folding signals in different genes

In addition, for each gene i we define the Selection Enrichment Score (SES) as:

$$\mathrm{SES_i} = \frac{\text{\# of clusters in gene i } / \text{ length of gene i}}{\mathit{total}\text{ \# of clusters } / \ \mathit{size}\text{ of genome}}$$

SESj values greater than 1 correspond to genes with a higher enrichment of clusters with respect to the entire genome. The results are summarized in tables T3 and T34:

	ancC	prM	E	NS1	NS2a	NS2b	NS3	NS4a	NS4b	NS5
**DENV1**	0	0.77	1.29	0.91	0.58	1.95	1.03	1.51	1.54	0.78
**DENV2**	0.91	0.99	1.05	1.18	1.67	1.19	0.84	0.41	1.04	0.86
**DENV3**	0.96	1.32	0.99	0.77	1.25	0.83	0.97	0.43	0.88	1.09
**DENV4**	1.81	1.24	1.04	0.58	0.94	0.78	1.16	1.21	1.03	0.85

Table T3. Enrichment of DENV genes with clusters of positions with a significant conservation of strong folding signals

	ancC	prM	E	NS1	NS2a	NS2b	NS3	NS4a	NS4b	NS5
**DENV1**	1.82	0.83	0.70	1.57	0.63	0.53	1.23	0.54	0.83	0.99
**DENV2**	0.81	1.12	1.03	0.79	0.85	1.06	0.97	1.09	1.12	1.08
**DENV3**	1.02	1.41	0.94	0.99	1.33	0.89	0.85	1.38	0.94	0.97
**DENV4**	0.45	0.94	1.16	1.18	1.19	0.79	0.84	1.23	1.05	0.92

Table T4. Enrichment of DENV genes with clusters of positions with a significant conservation of weak folding signals
